# NO_2_-Sensitive SnO_2_ Nanoparticles Prepared Using a Freeze-Drying Method

**DOI:** 10.3390/ma17153714

**Published:** 2024-07-27

**Authors:** Lin Liu, Jinbo Zhao, Zhidong Jin, Fei Liu, Dewen Zhao, Zhengyang Liu, Fenglong Wang, Zhou Wang, Jiurong Liu, Lili Wu

**Affiliations:** 1 Key Laboratory for Liquid-Solid Structural Evolution and Processing of Materials, Ministry of Education and School of Materials Science and Engineering, Shandong University, Jinan 250061, China; liulinsut@163.com (L.L.); jinzhidong0624@163.com (Z.J.); 202134170@mail.sdu.edu.cn (F.L.); 202214165@mail.sdu.edu.cn (D.Z.); fenglong.wang@sdu.edu.cn (F.W.); wangzhou@sdu.edu.cn (Z.W.); jrliu@sdu.edu.cn (J.L.); 2 School of Materials Science and Engineering, Qilu University of Technology (Shandong Academy of Sciences), Jinan 250353, China; zjbwll@163.com; 3 School of Mechanical Engineering, Shandong University of Technology, Zibo 255000, China; ytlyc@126.com

**Keywords:** SnO_2_, freeze-drying methods, electrical properties, NO_2_ sensors

## Abstract

The n-type semiconductor SnO_2_ with a wide band gap (3.6 eV) is massively used in gas-sensitive materials, but pure SnO_2_ still suffers from a high operating temperature, low response, and tardy responding speed. To solve these problems, we prepared small-sized pure SnO_2_ using hydrothermal and freeze-drying methods (SnO_2_-FD) and compared it with SnO_2_ prepared using a normal drying method (SnO_2_-AD). The sensor of SnO_2_-FD had an ultra-high sensitivity to NO_2_ at 100 °C with excellent selectivity and humidity stability. The outstanding gas sensing properties are attributed to the modulation of energy band structure and the increased carrier concentration, making it more accessible for electron exchange with NO_2_. The excellent gas sensing properties of SnO_2_-FD indicate its tremendous potential as a NO_2_ sensor.

## 1. Introduction

With the development of industry, global environmental pollution has become increasingly serious, and the World Health Organization (WHO) considers nitrogen dioxide (NO_2_) to be a serious pollutant [1]. NO_2_ is a significant source of global warming, haze, acid rain, and photochemical smog [2]. Moreover, NO_2_ has an impact on vegetation and crops by affecting plant growth efficiency and reducing crop yields [3]. On the other hand, NO_2_ is hazardous to human health, and high levels of NO_2_ inhalation can cause severe health risks such as pulmonary edema, breathing difficulties, and bronchospasm [4]. Long-term exposure to NO_2_ increases the risk of high blood pressure [4]. According to statistical analyses, each 10 μg/m^3^ increase in NO_2_ exposure increases all-cause mortality by 2%, acute lower respiratory disease by 6%, and chronic obstructive pulmonary illness by 3% [1,5,6]. Therefore, the development of sensors responding to low concentrations of NO_2_ is urgently demanded for improving the air environment and protecting human health.

Currently, the most common gas sensors are electrochemical sensors [7,8], solid electrolyte sensors [9], optical sensors [10,11], and semiconductor sensors [12,13]. Semiconductor sensors are widely used in the detection of toxic and hazardous gases owing to their low cost, high sensitivity, and good stability [14]. However, semiconductor sensors still have problems such as poor selectivity, high operating temperature, etc., which hampers their actual applications.

As a typical n-type metal oxide, SnO_2_ has excellent physical and chemical stability, with a low cost and non-toxic characteristics, which makes it widely used in gas sensors [15]. In recent decades, researchers have been devoted to tackling the aforementioned problems via multiple approaches for metal oxide semiconductor (MOS) sensors, including geometric structure modification [16], elemental doping [17], heterostructure construction [18,19], and noble metal loading [20]. Huang et al. prepared nanoflower-like Au/SnS_2_/SnO_2_ heterojunctions using a solvothermal method and in situ decoration. The response value to 8 ppm NO_2_ was 22.3 at 80 °C. These good gas-sensitizing properties were attributed to the formation of heterojunctions and the formation of more S vacancies, promoting more gas adsorption on the material surface [21]. Mnrugesh et al. synthesized p-Co_3_O_4_/n-SnO_2_ heterojunctions using a hydrothermal method. The prepared 10% Co_3_O_4_/SnO_2_ had a response of 88% at 150 °C for 100 ppm NO_2_ with good selectivity. The enhancement of the sensing properties was attributed to the formation of a potential barrier at the Co_3_O_4_/SnO_2_ heterointerface, the high specific surface area, and the increase in oxygen vacancy content [22].

Unfortunately, disadvantages still exist with the above modification strategies. Doping inhomogeneous elements into the MOS matrix will change the original crystal structure and increase surface defects [23]. The construction of heterojunctions via exogenous MOS or noble metals will increase the interfacial potential barrier, thereby increasing the baseline resistance and power consumption as well [24,25,26]. All of the above methods require the introduction of other elements into the MOS matrix, which increases the preparation cost and makes the production process more cumbersome. Moreover, freeze-drying treatment does not form a gas–liquid interface during the whole process, and the capillary force does not cause structural collapse. During the freeze-drying process, the material is first cooled below its freezing point, where the moisture in it freezes to form ice crystals. The formation and growth of ice crystals exert physical stresses on the surrounding material [27,28]. For semiconductor materials, this stress can lead to lattice distortions, which can introduce defects such as point defects, dislocations, and other defects, which, in turn, affect the electronic properties of the material. And the introduction of these defects can introduce new energy levels in the forbidden bands of semiconductors as trap energy levels or composite centers [29,30]. Hitherto, fewer studies have been reported on pristine MOS-based material sensors through freeze-drying treatments.

In this work, SnO_2_ nanoparticles were prepared using both a hydrothermal method and the following freeze-drying treatments. The results showed that the response value of SnO_2_-FD (886.2) to 10 ppm NO_2_ at 100 °C was 17 times higher than that of SnO_2_-AD (52.5), with a shorter response recovery time (74/27 s) and a low detection limit (1.69 ppb). The effect of the drying method on their gas-sensitizing properties was systematically investigated. The small particle size of the nanoparticles allowed a larger area to be in full contact with the target gas, which provided more active sites for gas adsorption. The enhanced performance is also attributed to the increase in adsorbed oxygen and the improvement of electronic structure. Therefore, this study paves novel ways for developing high-performance MOS-based sensors.

## 2. Experimental Section

### 2.1. Chemicals

Tin tetrachloride pentahydrate (SnCl_4_·5H_2_O, 99.0%), urea (CO(NH_2_)_2_, 99.5%), and ammonia solution (NH_3_, 25.0 ~ 28.0%) were purchased from Sinopharm Chemical Co., Ltd (Shanghai, China) and were used without further purification. Deionized water (DI) and absolute ethanol (C_2_H_5_OH, 99.7%) were also used in this work.

### 2.2. Synthesis of SnO_2_ Nanoparticles

SnO_2_ nanoparticles were synthesized using a facile hydrothermal method. In total, 2.35 mmol SnCl_4_·5H_2_O and 10.8 mmol urea were dissolved in a mixed solvent with a volume of 17.2 mL deionized water and 2 mL absolute ethanol with 15 min magnetic stirring. Then, 2 mL ammonia was added to the above solution. After another 15 min of magnetic stirring, the mixture was transferred into a 100 mL Teflon-lined stainless-steel autoclave and was maintained at 200 °C for 14 h. The white products were collected and washed with deionized water and absolute ethanol. Two drying methods were employed to remove solvents. One involved drying the products obtained via centrifugation at 80 °C. The other involved rapidly pre-freezing the products in liquid nitrogen after aging them in deionized water for 1 day to improve the stability of the samples and to form a more homogeneous ice crystal structure. And then, the samples were further freeze-dried at −50 °C for 2 days. The white powders obtained using the two methods were calcined at 500 °C for 2 h and were, respectively, named SnO_2_-AD and SnO_2_-FD.

### 2.3. Material Characterizations

The crystal structure of the samples was analyzed by X-ray diffraction analysis (XRD, DMAX-2500 PC, Tokyo, Japan) with Cu-Kα (λ = 1.5418 Å) from 10° to 90° with a scanning speed of 10°/min. The chemical compositions and the valence state of elements were characterized via an X-ray photoelectron spectrometer (XPS, AXIS Supra, Manchester, UK) with Al-Kα (hν = 1486.6 eV). The binding energy was calibrated using C 1s peaks at 284.8 eV. The morphology and microstructure of the samples were investigated by scanning electron microscope (SEM SU-70, Tokyo, Japan). The specific surface areas and pore size assignment of the samples were tested by a full-automatic specific surface and porosity analyzer (TriStar II 3flex, Micromeritics, Norcross, GA, USA) and separately calculated through Brunauer–Emmett–Teller (BET) and Barrett–Joiner–Halenda (BJH) methods. The electrical properties and carrier concentrations of the samples were measured by Hall Effect Measurement (HSM-5000, Seoul, Republic of Korea). The UV-vis spectra and band gaps of the samples were characterized via UV-vis diffuse reflection spectrum (Uv3600plus Shimadzu, Kyoto, Japan). The molecular structure of samples was analyzed by Raman spectroscopy (Thermo DXR2xi, Waltham, MA, USA) with a 1064 nm laser excitation.

### 2.4. Gas Sensing Performance Test

The gas sensors were fabricated using the prepared SnO_2_ materials. First, the prepared samples were dispersed in deionized water with a mass of 1:5 and thoroughly ground in a mortar to form a homogeneous paste. The paste was applied to an Al_2_O_3_ substrate with four electrodes printed on it and dried at 80 °C. This process was repeated five times to form a homogeneous sensitive film and heated in air for 10 h at 80 °C. Then, the substrates coated with the sensing layer were soldered to the pedestal and aged for one week at 3 V to ensure their stability. The gas sensing properties were measured with a WS-30B gas sensitivity instrument (Zhengzhou Winsen Electronics Co., Ltd., Zhengzhou, China). The target gases were injected into the test chamber via a syringe. Built-in fans in the test chamber rotated to bring the target gas into rapid and full contact with the sensor. R_a_ and R_g_ represent the stable resistance of the sensing material in air and after exposure to the target gas, respectively. The response value (S) is denoted by S = R_g_/R_a_ for oxidizing gases and S = R_a_/R_g_ for reducing gases. Response and recovery time are recorded as 90% time of total resistance changes in responding/recovering processes.

## 3. Results and Discussion

### 3.1. Characterizations

**Figure 1:**
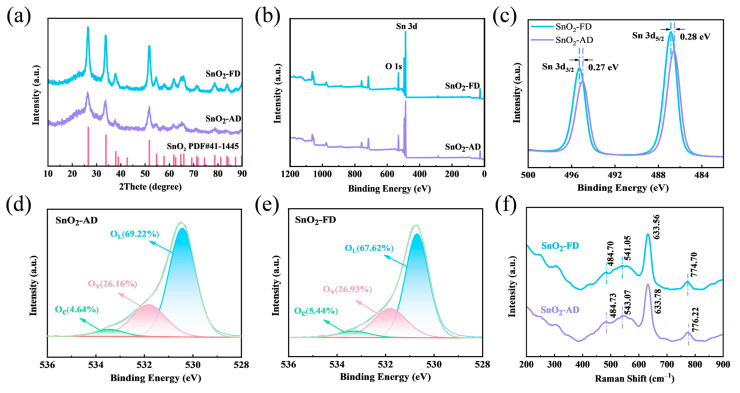
(**a**) XRD spectra of SnO_2_ samples. (**b**) XPS full-survey spectra and (**c**) Sn 3d XPS spectra of SnO_2_ samples. O 1s XPS spectra of (**d**) SnO_2_-AD and (**e**) SnO_2_-FD. (**f**) Raman spectra of SnO_2_ samples.

The crystal structure of SnO_2_ was measured by XRD as shown in Figure 1a. All diffraction peaks of SnO_2_-AD and SnO_2_-FD are in accordance with the tetragonal structure of SnO_2_ (JCPDS 41-1445). No other diffraction peaks appeared in the pattern, proving that the synthesized samples did not contain any other material phases. It can be observed that the SnO_2_-FD diffraction peaks are of higher intensity, indicating superior crystallinity compared to SnO_2_-AD [31]. Increased crystallinity means fewer grain boundaries, which are the scattering centers of carriers since the arrangement of atoms on the grain boundaries is different from that inside the grains [32]. On the other hand, grain boundaries are commonly accompanied by localized stresses and strains [33]. Therefore, the reduction in grain boundaries reduces carrier trapping and scattering at grain boundaries, thus improving carrier mobility of SnO_2_-FD [34]. The prepared SnO_2_ grain sizes can be approximately calculated using the Debye–Scherrer equation as indicated in Equation (1) [35]:

(1) $$D=\frac{0.9\lambda }{\beta \cos\theta }$$

where λ is the wavelength of the radiation (1.5418 Å), β is the half-height width of the peak, and θ is the Bragg diffraction angle. The average grain sizes are 10.3 and 9.0 nm, corresponding to SnO_2_-AD and SnO_2_-FD samples. SnO_2_-FD has a smaller grain size, and its grain size is close to twice the Debye length of SnO_2_ (3 nm) [36]. As we know, when the grain size of aerogels is nearly twice the Debye length, the size of the grains affects their electrical conductivity, that is, they are more likely to be activated for some nanometer effects [37]. Therefore, the depletion layer accounts for a large proportion of the particle volume, which is more favorable for exposing the SnO_2_ active surface and thus exchanging electrons with the target gas. Thereby, the response value and the response/recovery speed of SnO_2_-FD can be improved [38].

The chemical compositions and the valence state of elements were characterized via XPS. As shown in Figure 1b, the Sn and O elements are identified in the wide spectrum. The Sn 3d XPS spectrum of SnO_2_ is shown in Figure 1c, the two peaks at 486.52 eV and 495.03 eV corresponding to SnO_2_-AD are Sn 3d_5/2_ and Sn 3d_3/2_, respectively [39]. It can be observed that the Sn 3d_5/2_ and Sn 3d_3/2_ peaks of SnO_2_-FD are, respectively, shifted by 0.28 eV and 0.27 eV toward the high binding energy. Previous studies have shown that the total charge of an atom has a close influence on the chemical shifts of the peaks of the energy spectrum [40]. The SnO_2_-FD binding energy displays a redshift, indicating that more electrons are captured by the O_2_ molecules in air, resulting in a lower density of nearby electron clouds and an increase in the binding energy [41]. Figure 1d, e shows the O 1s XPS spectra. The peaks of SnO_2_-AD located at ca. 530.4, 531.8, and 533.4 eV correspond to lattice oxygen (O_L_), oxygen vacancy (O_v_), and adsorbed oxygen (O_c_), respectively [24,42]. It can be noted that the O_c_ and O_v_ contents of SnO_2_-FD are higher than those of SnO_2_-AD. The presence of O_v_ can supply more electrons and promote the formation of adsorbed oxygen ions [43,44]. On the other hand, O_v_ disrupts the metal oxide integrity and provides more active sites for target gas adsorptions and gas-sensitization reactions [45,46,47]. In particular, the increase in O_c_ may promote an alternative gas-sensitive reaction pathway for NO_2_ at the material surface [48,49,50].

The crystallography and structural features of SnO_2_-AD and SnO_2_-FD were investigated via a Raman system as shown in Figure 1f. The SnO_2_ lattice typically generates the following major vibrational modes [51]:

Γ = A_1g_ + A_2g_ + B_1g_ + B_2g_ + E_g_ + A_2u_ + 2B_1u_ + 3E_u_ (2)

where A_1g_, B_1g_, B_2g_, E_g_ are Raman active modes, A_2u_ and E_u_ are infrared active modes, A_2g_ and B_1u_ are inactive modes. The peak at around 633 cm^−1^ is assigned to the symmetric O-Sn-O vibration (A_1g_). The broadening of the A_1g_ peak of SnO_2_-FD indicates a reduction in its grain size [52]. The Raman peak at around 484 cm^−1^ corresponds to the shear vibration of the oxide (E_g_) [53]. And the Raman peak at around 776 cm^−1^ is due to the asymmetric O-Sn-O stretching (B_2g_) [54]. The Raman peaks of SnO_2_-FD all showed different degrees of blue shift, which might be caused by the increased content of oxygen vacancies [52]. The appearance of these Raman peaks indicates the tetragonal structure of SnO_2_. The peaks near 249 and 306 cm^−1^ are inactive Raman modes, which can be attributed to localized structural disturbances [55]. The enhancement of their strength is possibly due to structural defects introduced during the freeze-drying process.

**Figure 2:**
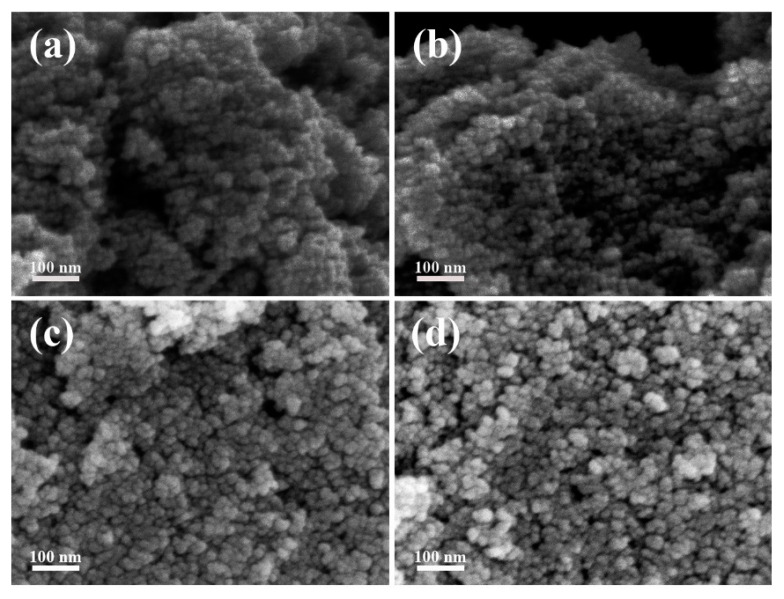
SEM images of (**a**,**b**) SnO_2_-AD samples and (**c**,**d**) SnO_2_-FD samples.

The morphology and microstructure of the samples were investigated by SEM as shown in Figure 2. It can be seen that both SnO_2_-AD and SnO_2_-FD are homogeneous nanospheres. It is indicated that the two drying methods have no significant effect on their morphology. The diameters of the SnO_2_ nanospheres are all approximately 10 nm, corresponding to the XRD results. This suggests that each SnO_2_ nanosphere is composed of a single crystal [56]. Moreover, such a small particle size gives them a larger specific surface area for full contact with the target gas [57]. The presence of abundant pore structures between the nanospheres further facilitates target gas diffusion.

**Figure 3:**
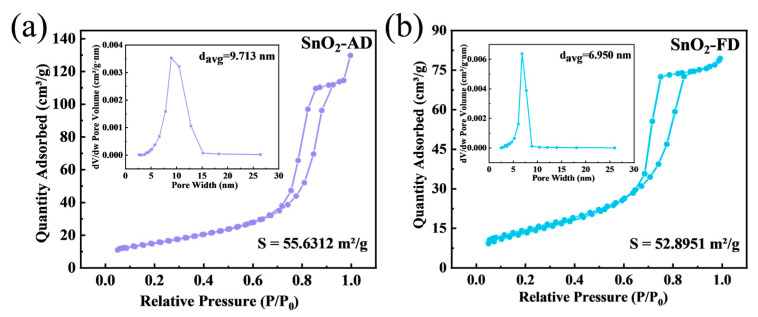
N_2_ adsorption–desorption isotherms and BJH pore size distributions (inset) of (**a**) SnO_2_-AD and (**b**) SnO_2_-FD.

To further analyze the specific surface area and pore size distribution of SnO_2_, N_2_ adsorption–desorption tests were performed as shown in Figure 3. The N_2_ adsorption–desorption isotherms of both SnO_2_-FD and SnO_2_-AD are of type IV mode with the type H2(b) hysteresis loop, indicating that both of them have mesoporous structures with similar hierarchical structures [58]. The specific surface areas of SnO_2_-AD and SnO_2_-FD are ca. 55.63 and 52.90 m^2^/g, respectively. The larger specific surface areas are attributed to the small particle size of SnO_2_ nanoparticles. This large specific surface area supplies more active sites for the adsorption of the target gas, which is positive for the surface of the gas-sensitive reaction, thus shortening the response/recovery time of the sensors and enhancing the response value [59,60]. As displayed in BJH measurement, the average pore sizes of SnO_2_-AD and SnO_2_-FD were calculated as ca. 9.71 and 6.95 nm, respectively. The smaller pore size of SnO_2_-FD indicates the presence of smaller primary particles formed, tightly aggregating to form smaller mesopores [61]. The pore size of the mesopore facilitates the adsorption and desorption of the target gas, thus effectively enhancing the gas sensing performance of SnO_2_ [62,63].

### 3.2. Gas Sensing Performance

**Figure 4:**
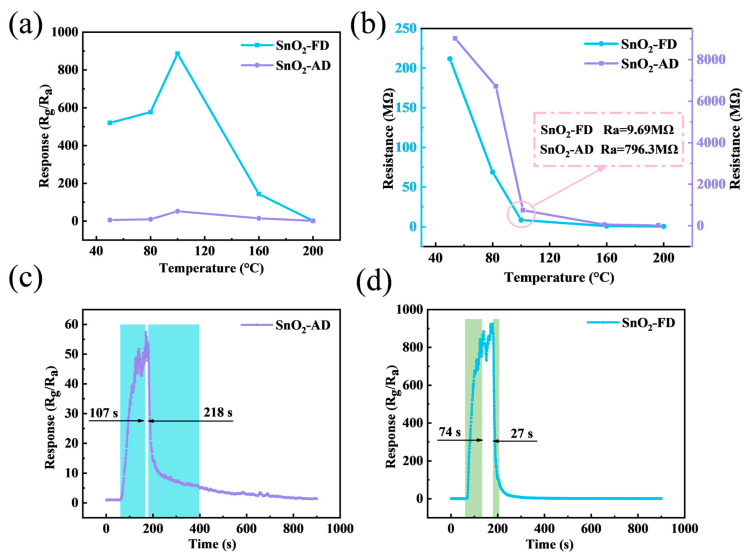
(**a**) The response of SnO_2_ sensors to 10 ppm NO_2_ under different operation temperatures. (**b**) The resistance of SnO_2_ sensors at various operation temperatures. The response/recovery curves of (**c**) SnO_2_-AD and (**d**) SnO_2_-FD sensors to 10 ppm NO_2_ at 100 °C.

The NO_2_ sensing characteristics of SnO_2_ sensors were investigated. The optimal operating temperature is an important indicator for evaluating the performance of gas sensors. Figure 4a shows the response of SnO_2_ sensors to 10 ppm NO_2_ under different operation temperatures. The response values of both SnO_2_-FD and SnO_2_-AD increase with increasing temperature and decrease after reaching a maximum at 100 °C. The reason is that the lack of thermal energy leads to gas adsorption that is weak or insufficient to overcome the energy barrier for gas-sensitive reactions at low temperatures, while the gas desorption rate is too fast for gas-sensitive reactions to occur at higher temperatures [64,65]. The response value of SnO_2_-FD (886.2) to 10 ppm NO_2_ at 100 °C is about 17 times higher than that of SnO_2_-AD (52.5).

Figure 4b illustrates the baseline resistance variation of SnO_2_-FD and SnO_2_-AD at various temperatures, and it can be observed that the baseline resistance of the SnO_2_ samples decreases with increasing temperature, exhibiting typical semiconductor characteristics [66]. Interestingly, the baseline resistance of SnO_2_-AD is about two magnitudes higher than that of SnO_2_-FD at the respective temperatures. This may be due to differences in carrier concentration. SnO_2_-FD has a higher concentration of carriers and therefore has a higher conductivity leading to a lower baseline resistance [67]. On the other hand, as an n-type semiconductor, the response value (S) of SnO_2_ to the oxidizing gas NO_2_ is defined by the ratio of the stabilized resistance (R_g_) exposed to NO_2_ to the baseline resistance (R_a_) in air. A small baseline resistance causes a more significant change in resistance, resulting in a larger response value [68,69]. The response/recovery curves of SnO_2_-AD and SnO_2_-FD sensors to 10 ppm NO_2_ at 100 °C are shown in Figure 4c,d. The response/recovery times of SnO_2_-FD are all shorter than those of SnO_2_-AD. In particular, the recovery time of SnO_2_-FD is 27 s profoundly lower than that of SnO_2_-AD (218 s), which is due to the increased porosity and the small particle size of SnO_2_-FD that promotes gas diffusion. To avoid errors due to serendipity, we performed two repetitive response recovery tests for SnO_2_-FD and SnO_2_-AD, respectively. As shown in Figure S1, the response/recovery times of SnO2-AD were 83/244 s and 96/202 s, whereas the response/recovery times of SnO_2_-FD were 71/43 s and 85/25 s, respectively. This indicates a significant improvement in the adsorption/desorption kinetics of SnO_2_-FD.

**Figure 5:**
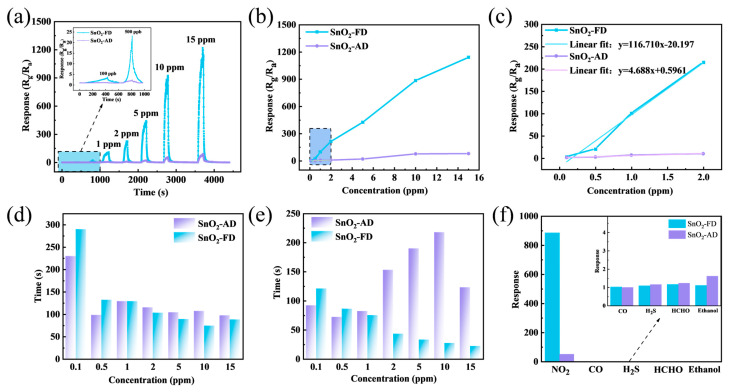
(**a**) Dynamic response curves of SnO_2_ sensors toward 0.1–15 ppm NO_2_ at 100 °C. (**b**) The response of SnO_2_ sensors toward different concentrations of NO_2_ at 100 °C. (**c**) The linear relationship between response value and NO_2_ concentration from 0.1 ppm to 2 ppm for SnO_2_ sensors. (**d**) The response time and (**e**) the recovery time of SnO_2_ sensors in response to NO_2_ with different concentrations. (**f**) The response of SnO_2_ sensors to 10 ppm NO_2_, 10 ppm H_2_S, 100 ppm CO, 100 ppm HCHO, and 100 ppm ethanol.

The dynamic response curves of SnO_2_ sensors toward 0.1–15 ppm NO_2_ at 100 °C are shown in Figure 5a. The response values of SnO_2_-AD and SnO_2_-FD increase continuously with increasing NO_2_ concentration, and the response value of SnO_2_-FD is much higher than that of SnO_2_-AD over the entire concentration range. It can be observed that there is still a significant response of SnO_2_-FD to 100 ppb NO_2_. Figure 5b records the response of SnO_2_ sensors toward different concentrations of NO_2_ at 100 °C. The response value of the sensor is basically linear with NO_2_ concentration, indicating its potential capability of quantitative NO_2_ detection. Figure 5c is a magnified image of the NO_2_ concentration in the range of 0.1–2 ppm in Figure 5b. As can be seen from Figure 5c, the SnO_2_-AD sensor response values are all below 10 when the NO_2_ concentration is less than 2 ppm, whereas the SnO_2_-FD sensor still has a high response value toward a low concentration of NO_2_, which is still as high as 214.9 at 2 ppm. A linear fit is performed for the response values versus the concentration of NO_2_ in this range. The slope of SnO_2_-FD (116.7) is 25 times higher than the slope of SnO_2_-AD (4.7), indicating that the presence of a trace amount of NO_2_ can cause a variation in the response value. Moreover, the regression value (R^2^) of SnO_2_-FD reached 0.975, indicating a favorable linearity, which is capable of providing accurate concentration measurements. Furthermore, the good linearity simplifies data analysis [70]. We can calculate the actual gas concentration from the response value of the sensor output by the known linear equation fitted [59,71]. The detection limit (LOD) of the sensor is predicted by Equation (3) [72]:

(3) $$\mathrm{LOD}=3\times \frac{\mathrm{rms}}{\mathrm{slope}}$$

where rms is the root mean square deviation of the baseline resistance and slope is the slope of the fitted line. The LOD of SnO_2_-AD is 127.53 ppb, and that of SnO_2_-FD is 1.69 ppb NO_2_. And the regression value (R^2^) of SnO_2_-FD amounts to 0.975, indicating a high reliability in practical applications.

Figure 5d,e show the response/recovery time of SnO_2_ sensors to NO_2_ with different concentrations. It can be observed that the recovery time of SnO_2_-FD exposed to high NO_2_ concentration is drastically shortened, and the response time is also reduced. The response/recovery time of a gas sensor is related to the diffusion rate of the gas and its surface reaction rate [73]. Its response/recovery at low concentrations is dominated by the effect of the gas diffusion rate [74]. The target gas concentration gradient at the sensor surface is quite low, resulting in a long response/recovery time [75,76]. The responses of SnO_2_ sensors to 10 ppm NO_2_, 10 ppm H_2_S, 100 ppm CO, 100 ppm HCHO, and 100 ppm ethanol at 100 °C are displayed in Figure 5f. The sensor is generally unresponsive to all gases except NO_2_, indicating that the sensor has excellent selectivity for NO_2_. The comparison of the performance of the SnO_2_-FD sensor in this work with the reported NO_2_ sensor is shown in Table 1. Compared to the reported NO_2_ sensor, the SnO_2_-FD sensor exhibits a high NO_2_ response value (886.2) and a short response recovery time (74/27 s) towards 10 ppm NO_2_ at 100 °C with an extremely low detection limit (1.69 ppb).

**Figure 6:**
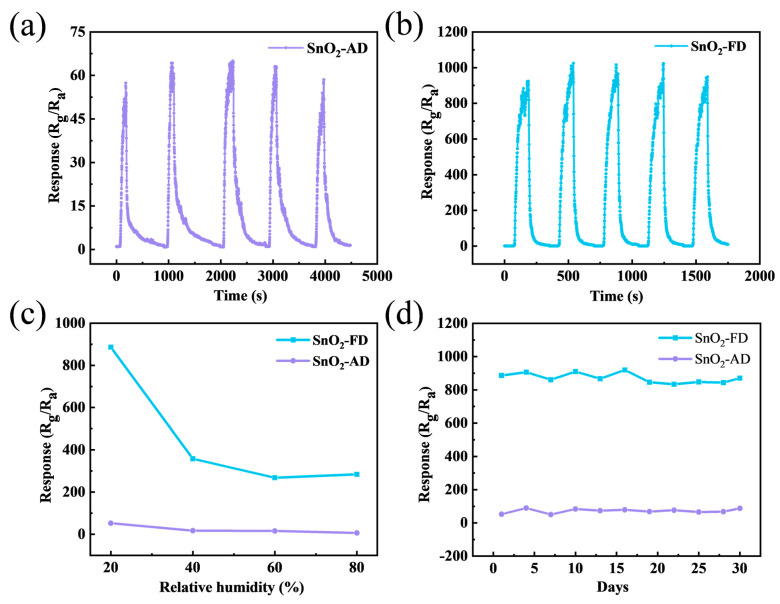
The stability of (**a**) SnO_2_-AD and (**b**) SnO_2_-FD sensors in response to 10 ppm NO_2_ at 100 °C in 5 cycles. (**c**) The response of SnO_2_ sensors under different humidity levels to 10 ppm NO_2_ at 100 °C. (**d**) The response change of SnO_2_ sensors in response to 10 ppm NO_2_ at 100 °C for 30 days.

Figure 6a,b show the stability of SnO_2_-AD and SnO_2_-FD sensors to 10 ppm NO_2_ at 100 °C in five cycles. The response values of the sensors remain essentially unchanged over the five cycles, indicating the good reliability of the sensors. Ambient humidity is a factor that must be taken into account in the practical application of gas sensors. The response of SnO_2_ sensors under different humidity levels to 10 ppm NO_2_ at 100 °C is shown in Figure 6c. The increased humidity leads to a reduction in the resistance of the material, as shown in Figure S2. It is attributed to the reaction of water molecules with adsorbed oxygen species on the surface of the material to form hydroxyl groups and release electrons into the conduction band of the material [81]. Moreover, the hydroxyl groups formed by water molecules can occupy the active sites on the material surface, which leads to metal oxide hydroxyl poisoning and inhibits gas adsorption [82,83]. On the other hand, the reaction of water molecules with adsorbed oxygen on the surface of the material generates a competitive relationship with the reaction of NO_2_ and adsorbed oxygen, which affects the gas-sensitive response of the sensor [84]. They stabilize at relative humidity up to 40 RH% and SnO_2_-FD still has a higher response value (284.29) at 80 RH% compared to SnO_2_-AD (6.41). Figure 6d shows the response change of SnO_2_ sensors to 10 ppm NO_2_ at 100 °C for 30 days. The response values of the SnO_2_-FD sensors are generally stable over a period of 30 days with an average value of about 871.86, indicating favorable long-term stability.

### 3.3. Gas Sensing Mechanism

**Figure 7:**
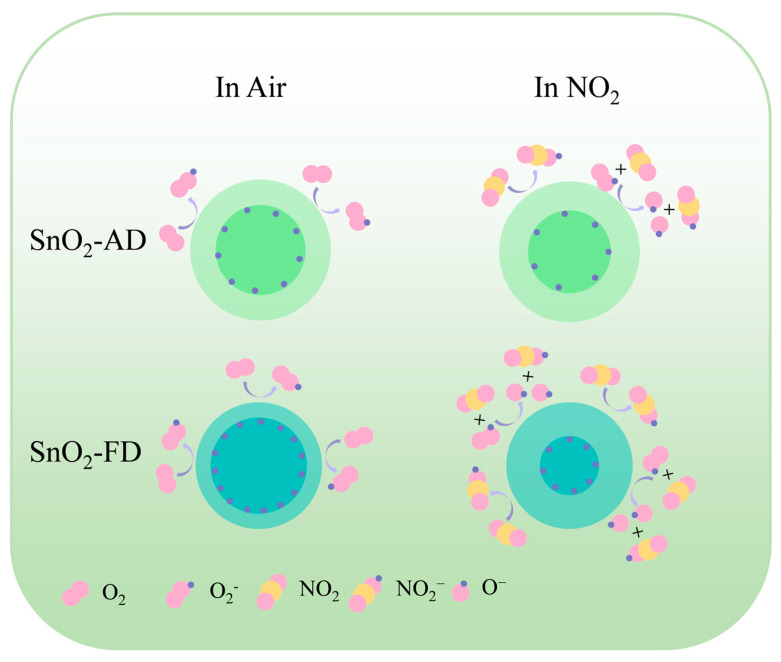
The NO_2_ gas sensing mechanism of SnO_2_-AD and SnO_2_-FD.

The gas sensing mechanism can be explained as the change in resistance of a semiconductor before and after exposure to a target gas, as shown in Figure 7. In air, oxygen molecules are adsorbed on the surface of the SnO_2_ sensor to capture its conduction band electrons to form reactive adsorbed oxygen species, resulting in an increase in SnO_2_ resistance [59]. Upon exposure of the sensor to NO_2_, NO_2_ further traps electrons in the conduction band of SnO_2_ due to its higher electron affinity than O_2_, leading to a further increase in its resistance [85]. On the other hand, NO_2_ reacts with adsorbed oxygen on the surface to form NO_2_^−^ resulting in a decrease in the content of O_2_^−^, which further robs the electrons in the SnO_2_ conduction band, leading to an increase in resistance [86]. The SnO_2_-FD and adsorbed oxygen content is higher than that of SnO_2_-AD as shown in Figure 1d, which may also be reasonable for why the response value and the response/recovery rate of SnO_2_-FD are much higher than those of SnO_2_-AD at a high NO_2_ concentration.

**Figure 8:**
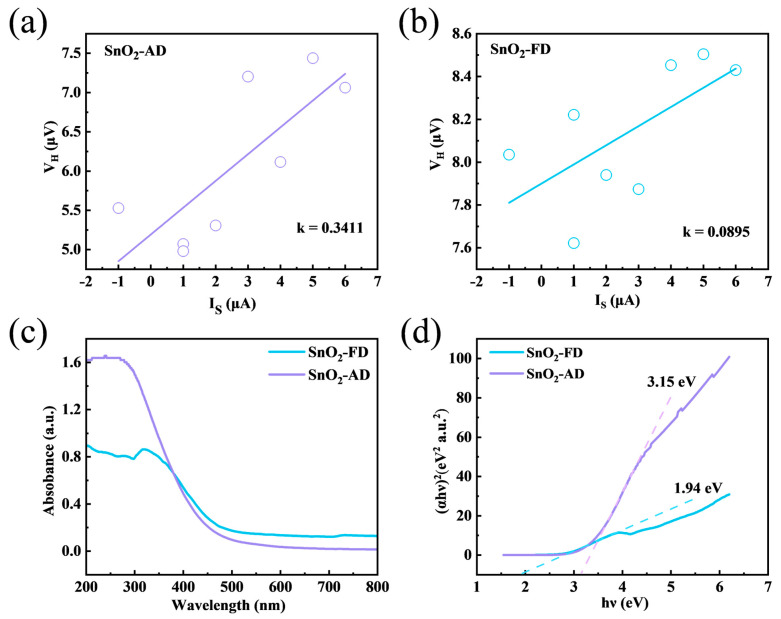
The V_H_−I curves measured by Hall Effect Measurement of (**a**) SnO_2_-AD and (**b**) SnO_2_-FD. (**c**) The UV−vis absorption spectrum of SnO_2_. (**d**) T−plots of (αhν)^2^ versus hν of SnO_2_.

In order to further understand the electrical properties, V_H_-I curves were tested using a Hall effect test system, as shown in Figure 8a,b, and the carrier concentrations were then calculated by Equation (4) [87]:

(4) $$n=\frac{\mathrm{IB}}{V_{H}\mathrm{ed}}$$

where I is the excitation current, B is the magnetic induction, V_H_ is the Hall voltage, and d is the material thickness. Here, V_H_/I can be expressed as the slope of the fitted straight line. The deviation of these dispersed points from the fitted straight line may be attributed to the non-uniform thickness of the coated gas-sensitive sensing layer, which results in a different concentration of electrons in each cross-section. During the measurement process, multi-point data were measured and fitted to minimize the error. The calculated carrier concentrations of SnO_2_-AD and SnO_2_-FD are 1.903 × 10^12^ and 7.251 × 10^12^ cm^−3^, respectively. According to the XPS results, the content of O/Sn in SnO_2_-AD (1.76) is higher than that in SnO_2_-FD (1.60), indicating that the intrinsic defects of n-type SnO_2_ recombine with oxygen, thus leading to a lower carrier concentration in SnO_2_-AD [88]. And the higher carrier concentration in SnO_2_-FD promotes a rapid gas sensing reaction [67]. To have a better understanding of the energy band structure, the UV-vis diffuse reflectance spectra of SnO_2_-AD and SnO_2_-FD were tested, as shown in Figure 8c,d. In the visible light wavelength range, the absorbance of SnO_2_-FD is higher than that of SnO_2_-AD, indicating that more carriers can be produced in SnO_2_-FD [89]. The UV absorption edge of SnO_2_-FD is redshifted; this is due to the straightforward electron transition between the valence bands and conduction bands, suggesting that the decrease in the band gap of SnO_2_-FD reduces the activation energy of the electron transition [90]. The band gap energies of SnO_2_-AD and SnO_2_-FD are ca. 3.15 and 1.94 eV, respectively, indicating that the preparation of SnO_2_ with the freeze-drying method significantly narrows the band gap. The reduction in the band gap may be due to the introduction of extensive defects [91]. This reduces activation energy for electron migration and allows NO_2_ to obtain electrons from the SnO_2_ conduction band more efficiently, thus increasing its response value and response recovery rate [40,72]. On the other hand, more electrons can be excited into the conduction band at a certain temperature, thus increasing the carrier concentration, which in turn promotes the electron transfer between the sensors and NO_2_ that facilitates the gas-sensitized reaction.

In this work, SnO_2_-FD has excellent NO_2_ sensing properties. First, the increase in chemisorbed oxygen content promotes an alternative reaction pathway for NO_2_ at high concentrations. Second, the SnO_2_-FD particle size is closer to the Debye length of SnO_2_, affecting its conductivity and facilitating the target gas contact with SnO_2_. In addition, SnO_2_-FD has a higher carrier concentration, which promotes electron exchange between the target gas and the sensing materials. Moreover, the band gap of SnO_2_-FD is drastically reduced, which lowers the activation energy of electrons transiting from the valence band to the conduction band and promotes the capture of electrons from the conduction band by the target gas, thus improving the response value of the sensor and the response/recovery speed.

## 4. Conclusions

Small-sized SnO_2_-FD particles prepared by hydrothermal and freeze-drying methods have good gas-sensitive properties for NO_2_ at lower temperatures. The SnO_2_-FD sensor exhibits an ultra-high response (886.2) with a short response recovery time (74/27 s) for 10 ppm NO_2_ at 100 °C. Moreover, the sensor exhibits an extremely low detection limit, good selectivity, and humidity stability. The SnO_2_ prepared by the freeze-drying method exhibits a significantly shortened band gap and increased carrier concentration, as well as a reduced particle size of SnO_2_ particles. This study provides a new idea for research on semiconductor gas-sensitive material preparation methods.

## Figures and Tables

**Table 1:** The NO_2_ sensing performance of reported sensors and this work.

Materials	Concentration (ppm)	S (R_a_/R_g_)	T (°C)	t_res_/t_rec_ (s/s)	LOD (ppb)	Ref.
In_2_O_3_/GO	40	78	225	106/42	-	[77]
NiCo_2_O_4_/n-WO_3_	20	153.7	150	13/16	52	[78]
Ce/ZnO	10	34.3	250	168/198	1.4	[50]
Rh/ZnO	10	36.17	150	32/512	50	[48]
Au/SnS_2_/SnO_2_	8	22.3	80	174/359.6	-	[21]
In_2_O_3_/MoS_2_	20	80.83	RT	152/179	8.8	[79]
Sn/In_2_O_3_	1	44.6	90	106/85	-	[80]
SnO_2_-FD	10	886.2	100	74/27	1.69	This work

## Data Availability

Data are contained within the article.

## Supplementary Materials

The following supporting information can be downloaded at: https://www.mdpi.com/article/10.3390/ma17153714/s1, Figure S1. The repetitive tests on the response-recovery of SnO_2_-AD and SnO_2_-FD to 10 ppm NO_2_ at 100 °C. Figure S2. The baseline resistance of SnO_2_-AD and SnO_2_-FD sensors at different humidity.
