# Supplementary material for: NO2-Sensitive SnO2 Nanoparticles Prepared Using a Freeze-Drying Method
Source: Materials (Basel). 2024 Jul 27;17(15):3714. doi: 10.3390/ma17153714 (PMC11313386; doi:10.3390/ma17153714)
Supplement: Supplementary file 1 [file materials-17-03714-s001.zip › materials-3106084-supplementary.pdf]

Supporting Information

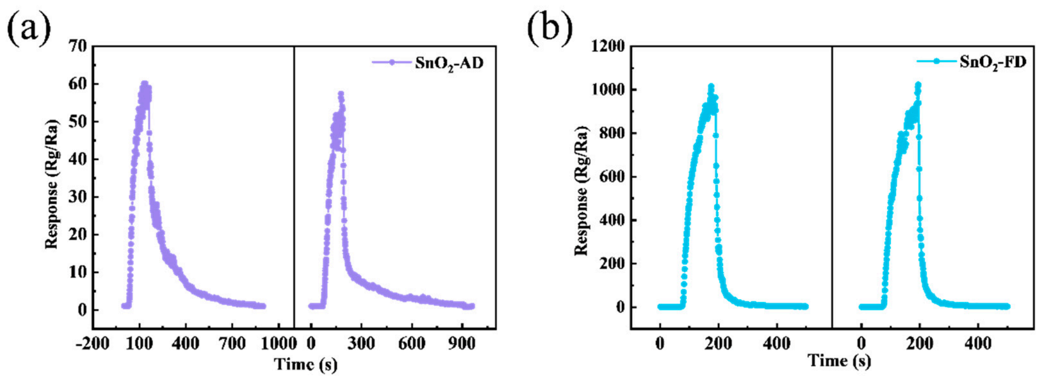

Figure S1. The repetitive tests on the response-recovery of SnO_2-AD and SnO_2-FD to 10 ppm NO_2 at 100°C.

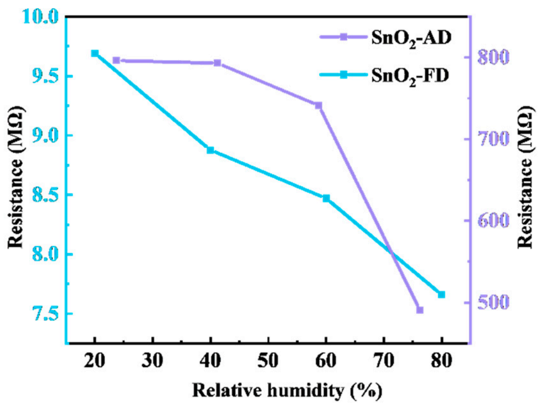

Figure S2. The baseline resistance of SnO_2-AD and SnO_2-FD sensors at different humidity.
